# Remarks on Abstinence; on Insufficient Nourishment; and Their Dangers

**Published:** 1831-04

**Authors:** P. A. Piorry


### ABSTINENCE.

### *Remarks on Abstinence; on insufficient Nourishment; and their Dangers.* By P. A. Piorry.*

Amongst the precepts which have been confirmed by age, *the utility of abstinence in acute diseases is one of the most incontestable:* but still, if we extend the application of this rule beyond proper bounds, the most dangerous errors may be committed; and there is reason to believe that this is sometimes the case with respect to the regimen imposed upon individuals labouring under disease. One fact should be impressed upon our minds: that the vital fluids play a very important part in the phenomena of life, and that any alteration which takes place in them must be productive of serious consequences. They may be modified in quantity, or in the proportion of their elements, or in the composition of the

* Journal Hebdom. No. 82.—Although, in this paper, M. Piorry combats many practical errors which are chiefly peculiar to the French school, and especially to the disciples of Broussais, it will be found to contain various hints, that many English physicians will attend to with advantage.—Editor.

materials of which they are composed: the blood, the principal source of the secretions, the constant stimulant of the organs, the vehicle of heat, and perhaps of vitality, is susceptible of these various alterations; and the inconveniences to which such alterations give rise are serious in proportion to the importance of the phenomena over which the blood presides. To repair the losses which the blood suffers, and to remedy the lesions which modify it, most of the organs of the body combine their influence. It appears that the ultimate use of all the viscera of the animal body, is to form a fluid capable of stimulating the nervous system, and, consequently, to support the powers of life: but it is alimentation which supplies them with the indispensable materials, and these instruments, so wonderfully perfect, and so curiously combined, would be reduced to impotence, if assimilable principles were not submitted to their reparative action. It must also be remarked, that our organs suffer as much from the want, as from the excess, of their natural stimuli. If blood is no longer carried to the brain, symptoms arise analogous to those of cerebral congestion; muscles, which remain inactive, sometimes become painful; the eye often remains inflamed in a dark place, and is cured by exposure to the light of day; when the stomach is deprived of food, severe pains arise in it, and obstinate vomitings occur. Let us inquire, then, from observation and reasoning, what are the results of rigid abstinence and insufficient alimentation, 1st, upon the blood and muscles; 2d, upon the heart; 3d, upon the lungs; 4th, upon the digestive tube, and the viscera connected with it; 5th, upon the nervous system.

1st. *Influence of abstinence upon the blood and muscles.* One of the first effects of rigorous and prolonged abstinence, is doubtless a diminution of the quantity of the blood, and an alteration in the proportions of its elementary principles. Experiments have been made upon this subject, which are extremely interesting. The quantity of fibrin in the blood is lessened in proportion as abstinence from food is prolonged, (Collard de Martigny;) the albumen appears, under the same circumstance, to be proportionately increased; its mass, however, remains the same. The volume of blood diminishes, and it is only by comparing the respective proportions of the fibrin and albumen, that we find the latter is become more abundant. The muscles soon lose their size and firmness. Fifteen days' rigorous abstinence are sufficient to render the muscles of the limbs thin, soft, easily lacerable, and very feeble. Their constituent principles are absorbed, in order to repair the loss sustained by the blood. The muscles of the trunk are reduced to a state of atrophy, and probably also the muscular fibres of the viscera. There is every reason to believe that the other organic elements are also partially absorbed, but perhaps less evidently and less rapidly, in the other tissues, and even in the fat and the fibrin of the muscles. Some persons would perish from hunger before they were emaciated: but, as a consequence of the deprivation of food, a deficiency arises in the circulatory apparatus, and, as experience shews that absorption is increased in proportion as blood is lost, (MAGENDIE,) we must admit this re-absorption theoretically; and it is indeed demonstrated by various pathological facts. It must not be forgotten, however, that this increased absorption would not be greater in a diseased than in a healthy tissue; and that we should in vain endeavour to procure by abstinence the total absorption of any abnormal production. We should no more succeed in such an attempt, than we should in causing a muscle to disappear entirely by starvation.

The quantity of blood is but little diminished during the first days in which a patient is deprived of food, because the various organs furnish materials to the blood, and fluids which are drank, and the liquids taken up by the pulmonary and cutaneous absorption, restore to it a part of the elements it has lost. But we soon witness the same phenomena which occur after severe hemorrhage: the tissues lose their natural colour; the lips, tongue, and conjunctive membrane of the eye, become paler; the veins are lessened in size, and contain but little blood; the circulation in them is slower than ordinary, as has been already explained;* the arteries beat with but little power, and hardly raise the finger which presses upon them; the volume of the heart is diminished; the lungs give a very clear sound; the liver shrinks. All these phenomena occur because the proportion of blood contained in the viscera is gradually diminished. The effects, then, of prolonged abstinence, as many cases have proved, are similar to those produced by loss of blood, and the symptoms which are thus produced are also similar: weakness, difficulty in moving, diminished energy of the various functions, frequent syncope when the head is raised, and death, from want of a due degree of cerebral excitement, constitute the usual succession of the subsequent phenomena. It is in this manner that many patients perish, either when disease of the alimentary canal prevents the process of assimilation, or when

* De la Circulation Veineuse, considerée sur le Rapport du Diagnostic.—Journ. Hebdomadaire, t. i. p. 442.

abundant evacuations have greatly diminished the reparative principles, or when a rigorous and prolonged abstinence has been imposed.

To deprive a patient of food for a long time, is to take from him a large quantity of blood. It would frequently be more prudent to have recourse to venesection, and at the same time allow moderate nourishment, than to inflict too severe a regimen for a long period. The one, no doubt, produces temporary debility; but it may be repaired by a suitable quantity of food: the other exhausts the patient, and is more dangerous, because its effects are slower and more insidious. Certain individuals have fallen under my observation, who were scarcely weakened by phlebotomy; but a few days' abstinence reduced them to a state of anemia. Loss of blood may be carried to a great extent, when the alimentary canal is capable of producing plenty of chyle; but it is much to be feared when the digestive process is obstructed. Attempts are made to cure consumption or cancer, by diminishing the quantity of food; but do we not perceive that diarrhœa, perspirations, and hemorrhagies, exhaust the reparative fluids. This severe regimen, by lessening the means of reparation, must greatly lessen the quantity of blood, and hasten the fatal moment. Irritation is much feared, but we should be still more apprehensive of inanition, and its terrible consequences. Rigorous abstinence destroys a dog in twenty-five or thirty days, and yet our patients are sometimes made to submit to it for months! It is to no purpose that pure water, or gum or sugar dissolved in water, emulsions, or lohocks, are given: they do not constitute sufficient nourishment for a sick man, any more than for a healthy one. Let an animal be submitted to such regimen, and his existence will soon terminate: the dogs which M. Magendie fed with sugar, oil, butter, and gum, died in about thirty days. If we wish to prolong life in an incurable disease, we must give chyle and blood to the system; or, in other words, we must give food.

Sometimes a cure wiil be effected by the efforts of nature, in cases which were considered irrecoverable. It cannot be imagined that it is a likely mode to cause the approximation of the parietes of an internal ulcer, or to make it cicatrize, by producing, by abstinence, a void in the vessels of the thorax, or a diminution in the volume of the organs. It must be admitted, that the blood is deteriorated in quality, in consequence of the absorption of the fluids contained in abscesses and cancerous parts; and it seems clear that the presence of chyle in the vessels will render absorption less rapid, and in part compensate for the evil effects of the absorption of the morbid secretions.

2. *On the effects of abstinence upon diseases of the heart.* Under the influence of abstinence, the heart, like all other muscles, is diminished in volume. Upon this fact is founded the practice of VALSALVA, which appears to be chiefly indicated in hypertrophy of the ventricles, provided it is not complicated with contraction of the orifice. A diminished quantity of food cannot dispel a scirrhous contraction, or ossification: it would probably, in some such cases, produce very mischievous effects. It is not without advantage that the muscular fibres are hypertrophied, when there exists any material obstacle to the circulation of the blood. The power becomes more active in proportion to the increased resistance it has to overcome. The obstacle may not be thus completely conquered, but it will be in part: that is to say, a large and powerful heart will impel the blood through a contracted, but still to a certain degree elastic, opening; whilst a small and feeble heart would not be capable of producing a similar effect. It is not the violent motions of the heart which are most frequently dangerous, but the organic alteration which gives rise to them. To diminish the size of the heart, is not the capital indication: our object would be to proportion the diameter of the arteries, and the openings of the heart, to the quantity of blood which is to traverse them, and to the demands of the organs whose actions the blood supports; but, unfortunately, art supplies no means of dilating the orifices or arteries: we are, therefore, compelled to diminish the mass of blood, and to insist upon a perfect state of repose on the part of the patient. By these means, there will be less blood to pass through the orifices, and the system in general will require a lesser quantity of this reparative fluid. To take away rapidly a certain quantity of blood may, therefore, be useful; but to diminish the quantity of fibrin which it contains, and to impoverish its quality, is irrational. Defective nutrition, and general debility, must be the inevitable results, and the fatal termination of the malady will be hastened. It is mechanically that general bleedings are useful in such cases, and therefore they are much to be preferred to the slow and gradual evacuations of blood from the bites of leeches. Rigorous abstinence, which deprives the blood of its reparative principles, which very slowly diminishes its mass, and which exercises the most prejudicial influence upon the digestive canal, cannot be substituted for the general abstraction of blood. It is to be remarked, also, that hypertrophy exists in two very different conditions. Sometimes the muscle is firm, and resists moderate pressure: such is the case in young subjects and robust men: a heart in this state is capable of contracting with energy. But at other times it is soft: a finger passes easily through its parietes: we find this condition of the viscus in old and feeble persons, and after procrastinated disease.* Here the heart acts feebly. In these two cases, the treatment must greatly differ. If it be true that in the first case, supposing that there exists no mechanical obstruction, rigid abstinence may be useful; in the second, wine and nourishing diet will be of the utmost advantage. Unfortunately the diagnosis is not yet sufficiently established to enable us, during life, to ascertain with certainty these different states of the heart. Auscultation has promised, upon this point, more than it has performed. The sounds of the heart are very deceptive; percussion is insufficient, and the functional symptoms are not to be relied upon. But there are, at least, probable grounds upon which we may act; and the practice of a wise physician, daily observation, and prudent and judicious percussion, may lead the steps of the practitioner, who would be misled by following an exclusive mode of treatment, and by always imposing rigorous diet and antiphlogistic remedies for every disease of the heart.

Simple dilatation of the heart, whether on the right or left side, does not require a complete privation of food: it cannot be a likely mode to produce contraction of the heart, to render it less firm and powerful. Prolonged abstinence from food produces, however, this effect upon the muscles of the nutritive functions, as well as upon those which regulate the locomotive powers. Neither can it be advantageous to diminish the volume of the abdominal viscera, in order to remedy an augmentation of the heart. The diaphragm, less firmly supported, would be less capable of resisting the tendency of the heart to dilate more and more. Now abstinence enfeebles the abdominal organs, and diminishes the quantity of substances which they contain. Perhaps it will be said that, under such circumstances, the muscles of the parietes will contract in proportion to the diminished capacity of the intestinal mass: but will they contract with energy, when continued abstinence has weakened their power?

Experience confirms this chain of reasoning. I have not observed, among the very numerous cases of dilatation of the heart at the Salpêtrière, that food produced any inconveni-

* At the Salpêtrière, hypertrophied hearts are usually found very soft in texture.

ence; and I may even say, with M. Merat, that I have known it relieve the sufferings of patients afflicted with aneurism. So far from believing that dilatation of the heart demands abstinence from food, it appears to me that, in general, in such cases, good nourishment is required; and if, at the same time, the organ is softened and thin, the most nutritive viands will be the most proper. The gastric organs will derive more fibrin from animal food than from gum or watery vegetables; and it is fibrin we require to give consistence to a softened muscle. I believe in the styptic action of carbonate of iron, or the mineral waters, and in the tonic powers of quinine; but I confess that I consider the best of all tonics to be nutritious food and wine of a good quality. Treat one emaciated convalescent with tonic medicines, and give him but little food: give another, in the same debilitated state, sufficient nourishment, and no tonics, and it will soon be evident which of the two will most quickly increase in muscular weight and strength.

I am by no means certain whether, in cases of aneurism of the large vessels, a vegetable diet, or rigid abstinence, is the most judicious means. The utility of bleeding is incontestable; but if it be useful to diminish the impulse of the blood, is it equally so to make it less consistent, paler, to diminish its quantity of fibrin, and to render it consequently less plastic? It is upon the plastic powers of the blood that we rest our hopes of prolonging life, and even of effecting a cure in some rare cases: and are not the means which increase the consistence of the blood most strongly indicated? such as substantial food and diluted alcohol.

Among the number of persons who have consulted me for palpitations of the heart, and difficulty in breathing, &c., and who presumed that they laboured under some organic affection of the organ, most had neither hypertrophy nor dilatation: no stethoscopic sound, no functional symptom, could lead to the suspicion of diminished capacity of the orifices of the heart. Many of these patients, and especially physicians, had confined themselves to vegetable diet in small quantities, without relief: they continued breathless from the least exertion, could with difficulty mount a staircase, and, from the continued increase of all their symptoms, the greatest mental alarm was produced. In consequence of not detecting any symptoms of organic lesion, the treatment was often completely changed. There appeared to be an analogy between the condition of these patients and that of a convalescent after procrastinated disease. In both cases, the most trifling motion produces a quickened and painful respiration, violent palpitations arise from the least moral impression, or upon the slightest attempt to ascend a declivity. The rapid improvement which takes place during convalescence after acute disease, as soon as the patient is freely nourished, was compared with the increasing languor of the persons who consulted me, and, relying upon the inferences which I drew, I prescribed substantial food in appropriate quantities, and its use was followed by decided amelioration of the symptoms. Abstinence from food will not remedy nervous palpitations: it frequently exasperates, and renders them intolerable. The following case is selected from many similar examples.

A lady, fifty years of age, saw her husband die suddenly by her side: the terror she experienced was almost beyond belief; she feared every moment that she was about to be attacked with apoplexy, and she also believed she was affected with organic disease of the heart. Violent palpitations, frequent determination of blood to the face, threatened her, in her own belief, with instant destruction. Four years' severe regimen, and repeated bleedings, produced no other effect than extreme debility. I found this patient, on consultation, almost exsangueous, with a thready miserable pulse, and the pulsation of the heart scarcely perceptible. Upon examination with the pleximeter, the heart was found to be of its natural size; its pulsations were regular, and no unusual sound was heard. Every tissue was pale and soft; the lips discoloured; tongue pale; eyes devoid of brilliancy, and the skin without warmth; her breathing was easy, but accelerated upon the slightest exercise; syncope occurred frequently during the day, and particularly when the patient first rose from her bed. The patient, constantly a prey to her fears, took the smallest possible quantity of nourishment, and insisted upon being bled the moment the slightest symptom arose which she thought to indicate determination of blood to the head. It was with the utmost difficulty she was persuaded that a totally different mode of treatment was required: she yielded, however, at length, and increased daily her quantity of food. In a short time she recovered, in a great degree, from the previous loss of blood. Her apprehensions were not entirely dispelled, but her palpitations were less violent, and exercise no longer produced them; syncope no longer occurred; the tissues manifested their natural colour; and every circumstance led to the inference that organic disease had never existed.

(To be continued.) p. 392.

